# Macroalgae-Fortified Sausages: Nutritional and Quality Aspects Influenced by Non-Thermal High-Pressure Processing

**DOI:** 10.3390/foods10020209

**Published:** 2021-01-20

**Authors:** Catarina Marçal, Carlos A. Pinto, Artur M. S. Silva, Carla Monteiro, Jorge A. Saraiva, Susana M. Cardoso

**Affiliations:** 1 LAQV-REQUIMTE, Department of Chemistry, University of Aveiro, 3810-193 Aveiro, Portugal; catarina.marcal@ua.pt (C.M.); carlospinto@ua.pt (C.A.P.); artur.silva@ua.pt (A.M.S.S.); jorgesaraiva@ua.pt (J.A.S.); 2 Irmãos Monteiro, S.A., Rua 5, Zona Industrial da Mota, Gafanha de Encarnação, 3830-527 Aveiro, Portugal; carlamonteiro@irmaosmonteiro.pt

**Keywords:** seaweeds, novel foods, sausages, frankfurters, nutritional, quality, shelf-life, microbial, high-pressure, storage

## Abstract

The present work evaluated the nutritional impact of macroalgae flours used as new ingredients in fermented sausages and the feasibility of using high-pressure processing (HPP) as a non-thermal pasteurization methodology to keep the quality attributes of the new food products. A commercial macroalgae mix was used in the formulation of new macroalgae-fortified meat frankfurter sausages (F-MFS), macroalgae-fortified vegetable frankfurter sausages (F-VFS) and in macroalgae-fortified traditional Portuguese sausage “chouriço” (F-TPS), overall incrementing the contents of Mg, K, Ca, Mn and Fe and decreasing the Na/K ratio. The application of HPP allowed extending the shelf-life of frankfurters by about 3-fold and improved the safety of “chouriço” along 180 days of storage, keeping its microbial load below the detection limit. The prevention of microbial growth in F-MFS and F-VFS was accompanied by pH stability of the products. In addition, no significant detriment on surface color and fatty acids was observed between pressurized and non-pressurized sausages, allowing consolidating the suitability of HPP in seaweed-fortified fermented sausages.

## 1. Introduction

Current modern lifestyles are characterized by lack of time and continuous stress, which favors unhealthy eating habits and unbalanced diets that are closely related to the main health problems of civilization such as diabetes, obesity, cardiovascular problems, decreased defenses, and lack of nutrients [1]. Despite this, consumers are becoming more aware and, therefore, food industries are striving to develop foods that are attractive to the consumers and rich in nutritional and/or functional terms [2]. In this context, algae, including marine macroalgae (i.e., seaweeds) are considered promising natural resources to be exploited as ingredients in foods in the future, not only because of their recognized richness in valuable nutrients (e.g., proteins, minerals and fibers) and in bioactive phytochemicals [3], but also due to the several advantages underlying their production, which contribute to green economy and sustainable development [4]. Because of that, new products containing macroalgae as a novel ingredient are exponentially being launched worldwide, including in Europe [5].

Sausages are traditionally made from ground meat, albeit many types and specificities are available worldwide, including even vegetarian products. To date, few authors focused on the impact of macroalgae-fortification on physicochemical features of sausages and other products, and most of them used macroalgae species characteristic of Eastern countries as new ingredients. In particular, Choi et al. concluded that the use of 3 macroalgae (*Laminaria japonica*, *Undaria pinnatifida*, *Hizikia fusiforme*) flour at 1% in low-salt sausages allowed to improve the cooking loss and the emulsion stability of the new products [6]. In turn, Cox et al. described the use of *Himanthalia elongata* (10–40%) in beef patties as a source of dietary fiber and antioxidants [7]. In fact, since meat products are important for a balanced diet, their fortification with macroalgae may represent a strategy to improve their nutritional or functional characteristics [8,9].

In addition to the raising numbers of new functional food products, the food industry pursues the application of new preservation methodologies that, besides safety, also guarantee improved sensory and nutritional characteristics of the products in comparison to the traditional processes [10]. In this context, high-pressure processing (HPP) appears as an emerging technique, with distinct food products already launched in the market [11], since it can inactivate microorganisms and extend the shelf-life of foods, when a pressure between 200–600 MPa is applied [12]. Unlike thermal processes, this technique allows the use of lower temperatures which leads to practically non-existent changes in nutritional properties and product quality [13]. Several studies claimed the efficiency of this technique in maintaining the characteristics of ready-to-eat products [14,15], some of them being already available in the market. Overall, the present study aimed to highlight possible nutritional changes of ready-to-eat sausages when using macroalgae prevalent in Europe as ingredients, as well as the impact of HPP on the quality and shelf-life extension of the novel food products. As the authors are aware, this is the first study reporting the effects of HPP on a heat-sensible food product fortified with macroalgae, allowing not only to ensure its microbial safety and raw-like characteristics but also to considerably extend the shelf-life, which can open the possibility of export this type of products to foreign geographies.

## 2. Materials and Methods

### 2.1. Chemicals

Light petroleum, hydrogen peroxide 30% (H_2_O_2_), sodium hydroxide (NaOH) were from Fisher (Waltham, MA, USA); nitric acid 69% (HNO_3_) was from Panreac (Barcelona, Spain); Ringer’s and plate count agar (PCA) medium was from Merck (Darmstadt, Germany); nonadecanoic acid 19:0 (Thermo Fisher Scientific, Kandel, Germany), fatty acid methyl esters (FAME) standards (Supelco. 37 Component FAME Mix, catalogue no. 47885-U, Supelco, Bellefonte, PA, USA). The powder mixture (*Ulva* spp., *Gracilaria* spp., *Fucus vesiculosus*) was from AlgaPlus Lda (Aveiro, Portugal).

### 2.2. Sausages

Plain sausages (control without macroalgae) and macroalgae-fortified sausages (new food products, with macroalgae) were produced by Irmãos Monteiro S.A. (a food company based in Gafanha da Encarnação, Aveiro, Portugal), following identical internal protocols which are subjected to confidentiality issues. Quantities of macroalgae powder were around 3% for meat-based sausages (2.6% in traditional Portuguese sausage (“chouriço”) (F-TPS) and 3.0% in meat frankfurter sausage (F-MFS), and of 7.8% in the vegetable frankfurter sausage (F-VFS). After preparation, the products were vacuum-packaged in flexible polyamide and polyethylene (PA/PE) bags and transported to the laboratory. There, several plain and macroalgae-fortified samples were stored at −20 °C for nutritional analysis (Section 2.3). In addition, just after arrival, a portion of macroalgae-fortified sausages were submitted to HPP (Section 2.4). Pressurized (HP) and non-pressurized (controls of HP samples) sausages were kept at a temperature of 4 ± 1 °C until analysis (for evaluation of the impact of HPP on quality parameters of macroalgae-fortified sausages during storage). The intervals of analysis for each sausage were primarily based on the specific shelf-life of plain sausages commercialized by Irmãos Monteiro S.A., and then readapted as appropriate, according to the gathered results, considering a maximum period of 180 days. In general, MFS and VFS were analyzed at the initial date (T0) and every 15 days up 30 (T15 and T30) or 45 days of storage (T45, just in pressurized frankfurters), while “chouriço” was analyzed every 30 days until 180 days (T0, T30, T60, T120, T150 and T180).

### 2.3. Nutritional Analysis of Plain and Macroalgae-Fortified Sausages

The protein content was estimated by elemental analysis of % nitrogen (N), through thermal conductivity using a Truspec 630–200–200 analyzer from LECO (St. Joseph, MI, USA), using the 6.25 conversion factor. For ash determination, the samples were dried at 105 °C (overnight), pre-incinerated for 20 min on a heating plate and then placed in the muffle, at a temperature of 550 °C for 6 h, followed by gravimetric quantification. The crude lipidic fraction was obtained from the Soxhlet extraction with light petroleum for 8 h, followed by filtration through a 0.2 µm nylon filter and drying of the solvent at a temperature below 40 °C, followed by gravimetric quantification.

The mineral composition was determined through acid digestion on microwave-assisted according to Monterroso et al. [16]. Briefly, a sample with up to 0.4 g was placed in the digestion vessel and 4 mL of HNO_3_ 69% (*w*/*w*) were added. The vessels were capped and placed in a microwave pressure digestor Speedwave MWS-3+ (Berghof, Eningen, Germany) and subjected two cycles to microwave radiation at 20 bar according to the following program: room temperature was raised first to 130 °C at 22 °C/min and 30% of irradiation power, then to 160 °C at 6 °C/min and 40% of irradiation power, for 5 min, and to 170 °C at 5 °C/min and 50% of irradiation power, for 5 min. The cooling process consisted of decreasing temperature first to 100 °C for 4 min and then to room temperature. The vessels were opened and 0.5 mL of H_2_O_2_ 30% (*w*/*w*) were added, being subjected to the second microwave digestion cycle. After cooling, acid digests were made up to 50 mL with Milli-Q water. Mineral content of Na, K, Ca, Mg, Fe, Mn, Cu and Zn was determined using an inductively coupled plasma mass spectrometry (ICP-MS), on a Thermo ICP-MS XSeries equipped with a Burgener nebulizer.

### 2.4. Effect of High-Pressure Processing on Macroalgae-Fortified Sausages

#### 2.4.1. HPP Conditions

The HPP treatments were carried out using a high-pressure equipment (Model 55 L, Hiperbaric S.A., Burgos, Spain). This equipment has a pressure vessel of 200 mm diameter and 2000 mm height. Tap water was used as pressurization fluid. Samples were treated at 600 MPa for 6 min at room temperature and were kept, together with non-pressurized samples (control for HPP) at 4 ± 1 °C until analysis (see period of analysis in Section 2.2).

#### 2.4.2. Microbial Analysis

At each time of analysis about 10 g of each sample was aseptically collected and homogenized in 45 mL of Ringer’s solution using the Stomacker (Milan, Italy) for 200 s (dilution 10^−1^). Successive dilutions were made from this dilution. Total aerobic mesophiles (TAM) were evaluated, while the value of 6.49 decimal logarithm of colony forming units per gram of sausage (log CFU/g sample) was considered to be the threshold for consumption. Thus, the PCA medium was used for quantification and the plates were incubated at 37 °C for 72 ± 3 h (ISO 4833: 2003). Plates displaying between 15–300 CFU were considered for the quantification.

#### 2.4.3. pH

Five grams of each sample were homogenized in 45 mL of boiled distilled water using an Ultra-Turrax at 12,000 rpm for 1 min. The mixture was then centrifuged at 6000 rpm for 10 min at 5 °C and the supernatant was filtered through G4 porous plate funnel. The pH reading was performed on the obtained filtrate using pH meter (XS instrument, Carpi, Italy).

#### 2.4.4. Surface Color

The color analysis was performed using the CIELab color space at 25 °C, by determination of the parameters L* (luminosity), a* (red/green color), and b* (yellow/blue color). A Konica Minolta CM 2300d spectrophotometer (Minolta Konica, Tokyo, Japan) was used and the CIELab parameters were determined using the original SpectraMagic™ NX Software v2.4, Konica Minolta, NJ, USA.

#### 2.4.5. Fatty Acids

The fatty acid profile was determined upon conversion of fatty acids to FAME, as previously described by Pinheiro et al. [17]. Briefly, to 300 mg of sample, 1 mL of the internal standard C19:0 (Thermo Fisher Scientific, Kandel, Germany) at 2 mg mL^−1^ in methanol, 0.7 mL of KOH (10 N) in water and 5.3 mL of methanol were added. The tubes were incubated for 1.5 h at 55 °C. Afterwards, 0.58 mL of H_2_SO_4_ (24 N) was added and these were placed at the same temperature for another 1.5 h. Finally, 3 mL of hexane was added, followed by mixing and centrifugation. The hexane fraction was recovered and injected on a gas chromatograph mass spectrometer GCMS-QP2010 (Shimadzu, Kyoto, Japan) equipped with an AOC-20i auto-injector and a DB-5 ms column (30 m × 0.25 mm diameter, 0.25 µm thickness). The equipment operated under the following conditions: initial temperature, 70 °C for 5 min; temperature gradient, 4 °C min^−1^; final temperature, 250 °C; temperature gradient, 2 °C min^−1^; final temperature, 300 °C for 5 min; injection temperature, 320 °C; split ratio, 100:0. Identification of FAME was obtained by co-chromatography with authentic commercially available FAME standards. Total FAME content was quantified by comparison with the internal standard.

#### 2.4.6. Statistical Analysis

Except for nutritional composition analysis (t-test), the statistical analysis of the remaining data was performed by Sidak multiple comparison tests. The statistical tests were applied using GraphPad prism 6.01 (GraphPad Software Inc.). A *p* value of 0.05 was considered statistically significant.

## 3. Results and Discussion

### 3.1. Impact of Macroalgae Fortification on the Nutritional Value of Sausages

Table 1 shows the details for important nutritional parameters of plain and macroalgae-fortified sausages. Moisture contents were in the range of 50–70% and decreased as plain vegetable frankfurter sausage (VFS) > plain traditional Portuguese sausage (TPS) > plain meat frankfurter sausage (MFS), tending to be lower in macroalgae-fortified products in comparison to the respective controls (i.e., plain products formulated without macroalgae). The levels of protein (20–22%) and fat (19–24%) in MFS and TPS were about 3 times that of VFS, while the latter was richer in carbohydrates (81% vs. 52–59%). Overall, the use of macroalgae as an ingredient in the sausages did not affect the levels of proteins and fats, but a clear trend to improve minerals was noted in the fortified products as compared to the plain sausages. This last fact is consistent with the high mineral richness of these macroalgae (20–33% of the dry weight (dw), as previously reported by our group) [18]. These results agree with those reported by other authors. e.g., when fortifying breakfast sausages with 1–4% of *Laminaria japonica*, Kim et al. found no variations in protein and total fat contents between the fortified products and the controls, while ashes levels were raised [19]. Likewise, López-López et al. described that the addition of 5.5% of *Himanthalia elongata* to functional frankfurters sausages caused a low impact on protein and fat contents, but significantly increased (*p* < 0.05) minerals [20].

As expected, Na and K were the main minerals in sausages, and Na/K ratios ranged between 1.8 to 3.6 in the plain products. These two minerals are also present in high amounts in macroalgae, but in general they are acknowledged for their richness in K and low Na/K ratio [21,22]. This fact is probably the cause of the decreasing trend of Na/K ratios in macroalgae-fortified sausages (range 3.0–1.6) compared to the respective plain products. Please note that this is a good indicator related to health issues, as low Na/K are recommended for promoting cardiovascular health [21]. Besides these minerals, others such as Ca and Mg were also relevant. Among the new products, the macroalgae-fortified meat frankfurter sausage (F-MFS) and the macroalgae-fortified traditional Portuguese sausage (F-TPS) were particularly rich in these minerals, reaching 28.6 ± 3.0 and 11.4 ± 0.1 mg/100 g (Ca) and 80.0 ± 1.3 and 61.5 ± 2.7 mg/100 g (Mg), respectively. Remarkably, these levels correspond to 3 and 2× for Ca and 3 and 4× for Mg, respectively, compared to the respective non-fortified products. Thus, our results suggest that the use of macroalgae as ingredients in meat-based sausages may represent a strategy to improve the levels of Ca and Mg in such type of food products.

### 3.2. Impact of HPP on Quality Parameters of Macroalgae-Fortified Sausages along Storage

The new food products i.e., macroalgae-fortified meat frankfurter sausage, macroalgae-fortified vegetable frankfurter and macroalgae-fortified traditional Portuguese sausage (F-MFS, F-VFS, and F-TPS, respectively) were pressurized at 600 MPa for 6 min at room temperature and effects on quality parameters (microbiological load, color, pH and fatty acids profile) were compared with control samples (i.e., not submitted to HPP) through storage at 4 °C.

#### 3.2.1. Microbial Analysis

**Figure 1:**
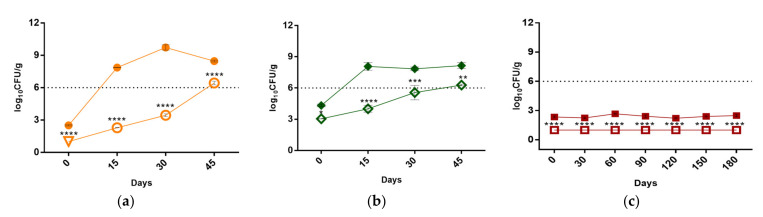
Evaluation of total aerobic mesophiles at 30 °C of (**a**) Macroalgae-fortified meat frankfurter sausage (non-pressurized—●, pressurized—○, and ▽—pressurized and below the detection limit), (**b**) Macroalgae-fortified vegetable frankfurter sausage (non-pressurized—◆ and pressurized—◇) and (**c**) Macroalgae-fortified traditional Portuguese sausage (pressurized and below the detection limit—□ and non-pressurized—■) during storage at 4 °C. *, **, ***, **** indicate significant differences between each pressurized and non-pressurized sample, *p* < 0.1, 0.01, 0.001 and 0.0001, respectively.

The evolution of the microbiological growth (TAM at 30 °C) in the sausages along storage time is shown in Figure 1. Notably, just after HPP, the F-MFS exhibited microbial loads below detection limits (1.0 log CFU g^−1^), contrasting with the control that had a TAM of about 2.5 log CFU g^−1^. Moreover, clear differences were registered with respect to the lag period to overpass the upper microbiological limit established (6.49 log CFU g^−1^): this was estimated to be < 15 days in control and close to 45 days in pressurized sample (Figure 1a). A similar trend was observed for the F-VFS, for which values of 4.0 and 6.3 log CFU g^−1^ and 8.1 and 8.2 log CFU g^−1^ were registered in pressurized sample and control at day 15 and 45 days, respectively (Figure 1b). Overall, microbiological results suggest that the application of HPP in the new macroalgae-fortified meat and macroalgae-fortified vegetable frankfurter sausages caused an increment of their shelf-life of about 3-fold.

As for F-TPS, the microbiological load of the products did not reach the upper microbiological limit value, ranging between 2.2–2.7 log CFU g^−1^ along the 6 months of storage in the control. Regardless of that, the results allow concluding that HPP improved the safety of the product, as the microbiological load in pressurized samples remained below detection limits along the period of analysis (Figure 1c).

In general, the herein gathered data are in accordance with those of previous studies, which showed the efficiency of HPP in decreasing the microbiological load of charcuterie food products. E.g., Kameník et al. reported that the application of HPP (600 MPa/5 min/room temperature) on cooked sausage lowered the TAM value, from 4 log CFU g^−1^ to 1.3 log CFU g^−1^, as monitored after 35 days of storage [23]. Similar effects were obtained by Botsaris et al., who reported a sharp decrease of total aerobic counts and a shelf-life extension of ham and meat frankfurters sausages by HPP application (600 MPa 3 min^−1^) on ham and meat frankfurters sausages [24]. Moreover, Garriga et al. described that HPP at 600 MPa (6 min at 31 °C) of cooked ham caused and extension of the shelf-life of the product (at 4 °C) from 30 to 90 days [25].

#### 3.2.2. pH

**Figure 2:**
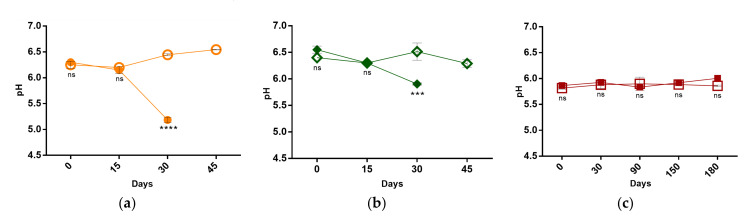
Evaluation of pH of (**a**) Macroalgae-fortified meat frankfurt sausage (pressurized—○ and non-pressurized—●), (**b**) Macroalgae-fortified vegetable frankfurt sausage (pressurized—◇ and non-pressurized—◆), (**c**) Macroalgae-fortified traditional Portuguese sausage (pressurized—□ and non-pressurized—■) during storage at 4 °C. ns indicate no significant differences, and ***, **** indicate significant differences between each pressurized and non-pressurized sample, *p* < 0.001 and 0.0001, respectively.

At T0, the pH values of F-MFS and F-VFS were higher than those of F-TPS (6.3–6.4 vs. < 6) (Figure 2), possibly because of inherent variations on ingredients and conditions of fermentation applied by the food industry in their manufacture. These products also differed greatly in pH variations over the storage period. In fact, when comparing the results from F-TPS with those of F-MFS and F-VFS, one must highlight the stability of the first in opposition to the significant decrement of pH of the macroalgae-fortified frankfurter sausages in the first month, reaching values of 5.2 ± 0.1 and 5.9 ± 0.0 at T30, respectively. Most likely, the observed pH lowering is associated with an increase in the microbial load of the products, as shown in Figure 1.

Notably, pH lowering was prevented by HPP in F-MFS and F-VFS, which also reflects the contribution of this technique to the maintenance of the quality of these types of sausages. In general, these results agree with the study of Devatkal et al. that registered a decrease in pH values in non-pressurized chicken nuggets after 30 days, contrasting with pH stability in HPP-treated samples [26]. In the same line, Han et al. reported a pH reduction from 6.4 to 5 in cooked ham after 30 days of storage, which was totally prevented by pressurization of the products at 600 MPa for 10 min at 22 °C [27]. However, Ruiz-Capillas et al. did not find significant pH differences between the pressurized and the control (pasteurized) products [28].

#### 3.2.3. Color Analysis

**Figure 3:**
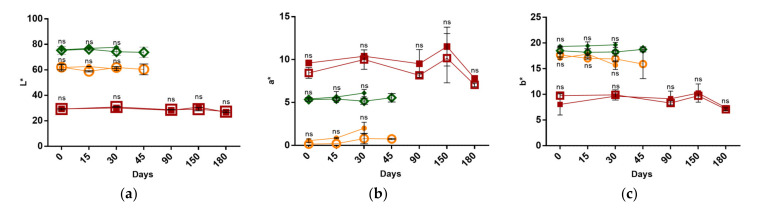
Color of fortified foods over time in different processing conditions (non-pressurized and pressurized). (**a**) L* (lightness) parameter, (**b**) a* (redness) parameter and (**c**) b* (yellowness) parameter of Macroalgae-fortified meat frankfurter sausage (pressurized—○ and non-pressurized—●), Macroalgae-fortified vegetable frankfurter sausage (pressurized—◇ and non-pressurized—◆), and Macroalgae-fortified traditional Portuguese sausage (pressurized—□ and non-pressurized—■) during storage at 4 °C. ns indicate no significant differences between each pressurized and non-pressurized sample.

The appearance and color of the surface of a food is a key factor determining consumers acceptance [29]. As can be observed in Figure 3, apart from oscillations that may be attributed to the high heterogeneity of the sausages, the results indicated no changes in the lightness (L*), redness (a*) and yellowness (b*) parameters of these food products throughout the tested period in non-pressurized macroalgae-fortified sausages, suggesting that the observed microbial growth and pH variations of frankfurters sausages (Figure 1a,b and Figure 2a,b) did not affect significantly their surface color. In addition, the gathered results demonstrated also that the applied HPP conditions did not significantly change the color parameters. These results are consistent with those obtained by Ruiz-Capillas et al., who observed no changes in color parameters in meat sausages, both in pressurized and control samples over time [28]. On the other hand, our results suggest that after long periods, F-TPS faded their reddish, a fact that is consistent with the results reported by O’Neill et al. and Cava et al. for vacuum-packed cooked ham [30,31].

#### 3.2.4. Fatty Acids Profile

The main fatty acids (FAs) (Table 2) detected in the three new macroalgae-fortified sausages were palmitic (C16:0), stearic (C18:0), oleic (C18:1), and linoleic (C18:2) acids, albeit their proportions differed significantly between meat-based and vegetarian samples. Meat products (i.e., F-MFS and F-TPS) were dominated by C16:0 and C18:0 and globally, saturated FAs (SFAs) accounted for more than half (51–59%) of their total FAs. In turn, the FA profile in F-VFS followed the sequence C18:2 > C18:1 > C16:0 > C18:0, and its unsaturated fraction represented about 70% of total FAs. Naturally, FAs profile of these sausages reflect their main ingredients, which in meat-based sausages are dominated by pork meat and backfat. In general, the FAs composition of meat products is in accordance with those reported by other authors for meat-based sausages [20,32].

When comparing the FAs profile of the macroalgae-fortified sausages at T0 and at T45 (non-pressurized frankfurters/pressurized frankfurters) and 180 days (for F-TPS), one may conclude that these were maintained along the storage period, thus suggesting that lipid oxidation reactions were negligible. A similar conclusion can be taken as regard to the products submitted to HPP, since except for polyunsaturated fatty acids (PUFA) of F-MFS, no significant variations were registered in the FA proportions between pressurized and non-pressurized samples. These results reinforce those previously obtained by Alfaia et al. for “chouriço” [33], who reported no influence of HPP on monounsaturated fatty acids (MUFA) and PUFA as well as the majority composition of FA.

## 4. Conclusions

The present study highlights the use of macroalgae as novel ingredients in fermented sausages, able to modulate the nutritional characteristics of the products, particularly with respect to improvement of their mineral profile. In addition, it reinforces the potential of HPP to be used in charcuterie products, particularly in macroalgae-fortified meat-based and vegetable-based frankfurters and in macroalgae-fortified traditional Portuguese sausage “chouriço”, by lowering of microbial load of the products without significant detrimental effects of color and FAs profile.

The incorporation of macroalgae in sausages did not contribute to a higher protein content, but proved effective in improving the mineral content, mainly Ca and Mg in F-MFS and F-TPS, by about 2- to 4-fold when compared to the control samples. In addition to the significant increase in these minerals, the seaweeds incorporation led to a decrease in the Na/K ratio in the new sausages.

The application of HPP in the different macroalgae-fortified sausages proved to be a method capable of inactivating microorganisms since it allowed extending the shelf-life for more 30 days in the F-MFS and F-VFS and improved the microbiological safety of F-TPS. This storage condition allowed also maintaining color parameters and fatty acid composition. Despite the promising results of the application of HPP, more studies such as estimation of liquid holding capacity, textural assays should be carried out to evaluate the maintenance of the physicochemical and sensory characteristics of products.

## Figures and Tables

**Table 1:** Nutritional composition of plain and macroalgae-fortified sausages.

(g/100 g)	Sample					
	MFS	F-MFS	VFS	F-VFS	TPS	F-TPS
Moisture	51.9 ± 1.1 ^a^	49.6 ± 1.1 ^a^	69.7 ± 0.4 ^a^	67.8 ± 0.7 ^b^	57.1 ± 1.7 ^a^	54.9 ± 2.8 ^a^
Protein	20.7 ± 0.8 ^a^	19.6 ± 0.2 ^a^	8.0 ± 0.1 ^a^	8.2 ± 0.2 ^a^	19.9 ± 0.9 ^a^	21.5 ± 2.2 ^a^
Total Fat	23.2 ± 0.02 ^a^	24.2 ± 0.1 ^a^	8.0 ± 0.1 ^a^	7.5 ± 0.01 ^a^	19.0 ± 0.6 ^a^	18.9 ± 0.1 ^a^
Carbohydrates	52.2 ± 1.0 ^a^	52.0 ± 0.02 ^a^	81.4 ± 0.3 ^a^	81.1 ± 0.5 ^a^	58.6 ± 0.4 ^a^	53.5 ± 2.3 ^a^
Ash	3.9 ± 0.1 ^a^	4.3 ± 0.2 ^a^	2.6 ± 0.2 ^a^	3.4 ± 0.5 ^a^	3.2 ± 0.4 ^a^	4.1 ± 0.4 ^a^
Minerals (mg/100 g)						
Na	1178.8 ± 8.2 ^a^	1153.2 ± 13.6 ^b^	604.1 ± 26.9 ^a^	674.0 ± 22.4 ^b^	589.1 ± 44.1 ^a^	758.3 ± 60.1 ^b^
K	329.5 ± 4.8 ^a^	387.9 ± 10.1 ^b^	308.1 ± 11.6 ^a^	379.0 ± 5.0 ^b^	325.7 ± 10.0 ^a^	488.7 ± 21.4 ^b^
Ca	12.5 ± 1.4 ^a^	28.6 ± 3.0 ^b^	22.5 ± 1.0 ^a^	27.5 ± 0.8 ^b^	4.33 ± 0.24 ^a^	11.4 ± 0.1 ^b^
Mg	19.0 ± 0.4 ^a^	80.0 ± 1.3 ^b^	6.33 ± 0.31 ^a^	12.4 ± 0.3 ^b^	20.8 ± 0.4 ^a^	61.5 ± 2.7 ^b^
Fe	1.00 ± 0.13 ^a^	2.88 ± 0.07 ^b^	<DL	0.98 ± 0.05	0.20 ± 0.03 ^a^	3.33 ± 0.12 ^b^
Mn	0.03 ± 0.05 ^a^	1.14 ± 0.05 ^b^	0.11 ± 0.01 ^a^	0.50 ± 0.01 ^b^	0.10 ± 0.01 ^a^	1.03 ± 0.06 ^b^
Zn	2.08 ± 0.01 ^a^	2.04 ± 0.02 ^b^	0.10 ± 0.01 ^a^	0.17 ± 0.06 ^a^	1.57 ± 0.06 ^a^	1.87 ± 0.12 ^b^
Cu	<DL	0.07 ± 0.03	<DL	0.03 ± 0.01	<DL	0.08 ± 0.02
Na/K	3.6	3	2	1.8	1.8	1.6

MFS—Plain meat frankfurter sausage; F-MFS—Macroalgae-fortified meat frankfurter sausage; VFS—Plain vegetable frankfurter sausage, F-VFS—Macroalgae-fortified vegetable frankfurter sausage; TPS—Plain traditional Portuguese sausage; F-TPS—Macroalgae-fortified traditional Portuguese sausage. DL—detection limit. Different letters (a, b) indicate significant differences (*p* < 0.05) between each plain and fortified product. The results shown were obtained from the mean of at least 3 independent assays.

**Table 2:** Fortified foods fatty acids composition (% of total fatty acids), at the different processing conditions (non-pressurized and pressurized).

	C-F-MFS		HP-F-MFS		C-F-VFS		HP-F-VFS		C-F-TPS		HP-F-TPS	
	T0	T45	T0	T45	T0	T45	T0	T45	T0	T180	T0	T180
Saturated												
C14:0	2.1 ± 0.01 ^aA^	2.4 ± 0.2 ^aA^	2.3 ± 0.3 ^aA^	2.5 ± 0.1 ^aA^	1.4 ± 0.04 ^aA^	1.3 ± 0.04 ^bA^	1.4 ± 0.1 ^aA^	1.3 ± 0.1 ^bA^	2.5 ± 0.01 ^aA^	2.6 ± 0.2 ^aA^	2.6 ± 0.1 ^aA^	2.4 ± 0.1 ^aA^
C16:0	35.1 ± 0.1 ^aA^	35.6 ± 0.1 ^aA^	34.5 ± 0.4 ^aA^	35.3 ± 0.1 ^aA^	12.5 ± 0.2 ^aA^	12.7 ± 0.5 ^aA^	12.7 ± 0.4 ^aA^	13.1 ± 0.4 ^aA^	31.6 ± 0.8 ^aA^	31.4 ± 0.8 ^aA^	29.9 ± 0.1 ^aA^	29.5 ± 2.8 ^aA^
C17:0	0.5 ± 0.01 ^aA^	0.6 ± 0.01 ^bA^	0.4 ± 0.01 ^aA^	0.6 ± 0.03 ^bA^	-	-	-	-	0.3 ± 0.1 ^aA^	0.4 ± 0.09 ^aA^	0.3 ± 0.1 ^aA^	0.4 ± 0.2 ^aA^
C18:0	20.4 ± 0.01 ^aA^	20.3 ± 0.3 ^aA^	20.8 ± 0.02 ^aA^	19.9 ± 0.1 ^aA^	7.3 ± 0.2 ^aA^	6.7 ± 0.8 ^aA^	6.8 ± 0.7 ^aA^	6.8 ± 0.6 ^aA^	17.2 ± 0.1 ^aA^	18.1 ± 0.7 ^aA^	17.8 ± 0.3 ^aA^	19.2 ± 1.1 ^aA^
C20:0	0.41 ± 0.01 ^aA^	0.5 ± 0.02 ^aA^	0.5 ± 0.03 ^aA^	0.5 ± 0.01 ^aA^	0.6 ± 0.1 ^aA^	0.6 ± 0.1 ^aA^	0.6 ± 0.03 ^aA^	0.6 ± 0.03 ^aA^	0.4 ± 0.04 ^aA^	0.4 ± 0.04 ^aA^	0.5 ± 0.2 ^aA^	0.4 ± 0.1 ^aA^
C22:0	-	-	-	-	3.5 ± 0.3 ^aA^	3.2 ± 0.3 ^aA^	3.5 ± 0.2 ^aA^	3.3 ± 0.1 ^aA^	-	-	-	-
C24:0	-	-	-	-	4.0 ± 0.3 ^aA^	3.5 ± 0.3 ^bA^	3.9 ± 0.2 ^aA^	3.5 ± 0.1 ^aA^	-	-	-	-
Unsaturated												
C16:1 (n-9)	2.1 ± 0.01 ^aA^	2.3 ± 0.1 ^aA^	2.1 ± 0.01 ^aA^	2.5 ± 0.1 ^aA^	0.7 ± 0.04 ^aA^	0.7 ± 0.1 ^bA^	0.7 ± 0.02 ^aA^	0.7 ± 0.01 ^bA^	2.7 ± 0.1 ^aA^	2.7 ± 0.2 ^aA^	2.7 ± 0.1 ^aA^	2.5 ± 0.1 ^aA^
C17:1 (n-9)	0.5 ± 0.01 ^aA^	0.6 ± 0.02 ^aA^	0.5 ± 0.01 ^aA^	0.6 ± 0.03 ^bA^	-	-	-	-	0.6 ± 0.1 ^aA^	0.6 ± 0.04 ^aA^	0.5 ± 0.02 ^aA^	0.5 ± 0.03 ^aA^
C18:1 (n-9)	25.3 ± 0.1 ^aA^	24.9 ± 0.3 ^aA^	25.5 ± 0.01 ^aA^	24.6 ± 0.2 ^aA^	28.8 ± 0.4 ^aA^	29.4 ± 0.1 ^aA^	28.8 ± 0.1 ^aA^	29.2 ± 0.2 ^aA^	26.0 ± 0.9 ^aA^	25.3 ± 0.6 ^aA^	26.0 ± 0.7 ^aA^	26.3 ± 0.2 ^aA^
C18:2 (n-6)	8.9 ± 0.1 ^aA^	8.2 ± 0.1 ^bA^	8.5 ± 0.04 ^aB^	8.6 ± 0.1 ^aB^	41.1 ± 1.2 ^aA^	41.9 ± 2.2 ^aA^	41.7 ± 1.6 ^aA^	41.5 ± 1.6 ^aA^	11.6 ± 0.3 ^aA^	11.4 ± 1.1 ^aA^	12.1 ± 0.01 ^aA^	11.7 ± 0.1 ^aA^
C20:1 (n-9)	0.9 ± 0.01 ^aA^	0.9 ± 0.1 ^aA^	0.9 ± 0.03 ^aA^	0.9 ± 0.01 ^aA^	0.02 ± 0.02 ^aA^	0.1 ± 0.02 ^aA^	0.06 ± 0.02 ^aA^	0.1 ± 0.1 ^aA^	0.6 ± 0.1 ^aA^	0.6 ± 0.02 ^aA^	0.5 ± 0.02 ^aA^	0.5 ± 0.2 ^aA^
C20:2 (n-6)	1.8 ± 0.01 ^aA^	1.7 ± 0.1 ^aA^	1.9 ± 0.04 ^aA^	1.9 ± 0.02 ^aA^	-	-	-	-	2.1 ± 0.1 ^aA^	2.0 ± 0.1 ^aA^	2.1 ± 0.1 ^aA^	2.0 ± 0.5 ^aA^
C20:3 (n-6)	0.7 ± 0.01 ^aA^	0.7 ± 0.1 ^aA^	0.8 ± 0.02 ^aA^	0.8 ± 0.02 ^aA^	-	-	-	-	1.3 ± 0.03 ^aA^	1.4 ± 0.2 ^aA^	1.5 ± 0.1 ^aA^	1.4 ± 0.3 ^aA^
C20:4 (n-6)	1.4 ± 0.01 ^aA^	1.4 ± 0.01 ^aA^	1.5 ± 0.1 ^aA^	1.4 ± 0.03 ^aA^	-	-	-	-	3.1 ± 0.1 ^aA^	3.1 ± 0.3 ^aA^	3.5 ± 0.02 ^aA^	3.2 ± 0.5 ^aA^
∑ SFA	58.4 ± 0.1 ^aA^	59.3 ± 0.6 ^aA^	58.5 ± 0.8 ^aA^	58.8 ± 0.4 ^bA^	29.4 ± 1.2 ^aA^	28.0 ± 2.0 ^aA^	28.8 ± 1.6 ^aA^	28.5 ± 1.3 ^aA^	52.1 ± 1.0 ^aA^	53.0 ± 1.8 ^aA^	51.1 ± 0.7 ^aA^	51.8 ± 4.2 ^aA^
∑ MUFA	28.8 ± 0.2 ^aA^	28.7 ± 0.5 ^aA^	29.0 ± 0.1 ^aA^	28.6 ± 0.4 ^aA^	29.6 ± 0.4 ^aA^	30.1 ± 0.2 ^aA^	29.5 ± 0.1 ^aA^	30.0 ± 0.3 ^aA^	29.9 ± 1.1 ^aA^	29.1 ± 0.9 ^aA^	29.7 ± 0.8 ^aA^	29.9 ± 0.6 ^aA^
∑ PUFA	12.8 ± 0.1 ^aA^	12.0 ± 0.3 ^aA^	12.6 ± 0.2 ^aA^	12.6 ± 0.1 ^aB^	41.1 ± 1.2 ^aA^	41.9 ± 2.2 ^aA^	41.7 ± 1.6 ^aA^	41.5 ± 1.6 ^aA^	18.1 ± 0.5 ^aA^	17.9 ± 1.6 ^aA^	19.2 ± 0.2 ^aA^	18.3 ± 1.4 ^aA^

C-F-MFS—Non-pressurized macroalgae-fortified meat frankfurter sausage; HP-F-MFS—Pressurized macroalgae-fortified meat frankfurter sausage; C-F-VFS—Non-pressurized macroalgae-fortified vegetable frankfurter sausage, HP-F-VFS—Pressurized macroalgae-fortified vegetable frankfurter sausage; C-F-TPS—Non-pressurized macroalgae-fortified traditional Portuguese sausage; HP-F-TPS—Pressurized macroalgae-fortified traditional Portuguese sausage. SFA—Saturated fatty acids, MUFA—Monounsaturated fatty acids, and PUFA—Polyunsaturated fatty acids. Different letters (a, b) in the same line for each sausage between initial and final time, indicate significant differences (*p* < 0.05). Different capital letters (A, B) in the same line for each sausage between different processing at the same time, indicate significant differences (*p* < 0.05). The results shown were obtained from the mean of at least 3 independent assays.

## Data Availability

The data that support the findings of this study are available within the article.
